# Different effects of polyethylene microplastics on bioaccumulation of three fungicides in maize (*Zea mays* L.)

**DOI:** 10.1007/s44297-024-00028-x

**Published:** 2024-05-21

**Authors:** Shuimin Qiu, Hongjian Shen, Jialu Song, Hua Fang, Yunlong Yu, Luqing Zhang

**Affiliations:** Institute of Pesticide and Environmental Toxicology, the Key Laboratory of Molecular Biology of Crop Pathogens and Insects, the Key Laboratory of Biology of Crop Pathogens and Insects of Zhejiang Province, College of Agricultural and Biotechnology, Zhejiang University, Hangzhou, 310058 China

**Keywords:** Adsorption, Bioavailability, Dissipation, Distribution, Hydrophobicity

## Abstract

Despite the ubiquity of microplastics (MPs) and pesticides in agricultural soils, the effects of MPs on the behavior and bioavailability of pesticides in soil–plant systems remain largely unknown. This study comparatively investigated the adsorption and dissipation of three commonly used fungicides (metalaxyl, azoxystrobin and tebuconazole) in soil as well as their accumulation and distribution in maize *Zea mays* L. with and without the amendment of polyethylene MPs (PE-MPs). The results showed that the adsorption of the fungicides to both MPs and soil was strongly dependent on their octanol/water partition coefficients (log*K*_ow_). The addition of 5% PE-MPs significantly increased the adsorption of the hydrophobic fungicides azoxystrobin and tebuconazole to soil due to their greater adsorption affinity to PE-MPs than to soil, while the effect was negligible in the case of the hydrophilic fungicide metalaxyl. The enhanced adsorption of azoxystrobin and tebuconazole to soil with the amendment of PE-MPs decreased their bioavailable fractions in soil, especially the concentration in in situ pore water, resulting in prolonged persistence in soil and reduced accumulation in maize plants. PE-MPs caused a greater reduction in the dissipation and bioaccumulation of tebuconazole than azoxystrobin, presumably because PE-MPs were more effective in promoting the adsorption of tebuconazole (with a higher log*K*_ow_) in soil. Comparatively, PE-MPs had little effect on the dissipation and bioaccumulation of metalaxyl since its bioavailability was almost unaffected. Our work provides effective information for the risk assessment of co-contamination of MPs and pesticides.

**Supplementary Information:**

The online version contains supplementary material available at 10.1007/s44297-024-00028-x.

## Introduction

Due to massive production and inadequate end-of-life disposal, large amounts of plastic waste are being discharged into the environment, where they are subjected to a cascade of natural fragmentation processes [1]. Consequently, microplastics (MPs, < 5 mm in diameter) are found ubiquitous in a range of environments, including oceans, lakes, and soils [2], raising concerns about their adverse ecological effects. Studies have documented that agricultural land is a major sink for MPs, which are mainly derived from mulch film, wastewater irrigation, organic fertilizer and compost, sewage sludge, etc. [3, 4]. It was estimated that up to 700,000 tons of MPs were discharged annually to agricultural soils in Europe and North America [5]. Although the MP abundance in agricultural soils might be higher than that in waters like oceans [6], a clear majority of the studies regarding the behavior and effects of MPs have focused on aquatic ecosystems, with much less attention paid to terrestrial ecosystems.

In addition to MPs, soil has the capacity of retaining other pollutants, such as pesticides, antibiotics, heavy metals, etc. Among all the xenobiotics in agricultural soils, pesticides are the most common contaminants due to their long-term and wide-scale application [7]. Because of their large specific surface area and strong hydrophobicity, MPs can adsorb various organic pollutants, including pesticides [8, 9], possibly changing their behavior in the environment. For example, low-density polyethylene (LDPE) microplastics reduced the soil adsorption capacity for imidacloprid and flumioxazin, and accelerated their degradation in soil [10]. Polyethylene microplastics (PE-MPs) were also found to reduce the adsorption capacities of atrazine and 4-(2,4-dichlorophenoxy) butyric acid to natural soil through dilution, potentially enhancing their mobility in soil [11]. On the contrary, a previous study reported that the presence of 1% MPs (polystyrene (PS), polypropylene and PE) increased the capacity of soil to retain phenanthrene and thereby reduced their mobility in soil [12]. PE-MPs addition was observed to inhibit the degradation of chlorpyrifos and ciprofloxacin in soils [13, 14]. The MP-induced alterations in the behaviors of pollutants likely affect their bioavailability and bioaccumulation in soil ecosystems. Several studies have found that MPs could enhance the accumulation of organic pollutants by soil fauna via vector effects [15, 16]. Although MP ingestion partially (8.7 ~ 26.1%) contributed to bioaccumulation, the addition of LDPE-MPs significantly reduced the freely dissolved concentrations (*C*_free_) of polychlorinated biphenyls (PCBs) in soil and thus decreased their accumulation in the earthworm *Eisenia fetida* [17]. Xu et al. [18] found that PS MPs reduced the uptake of phenanthrene by soybean seedlings. Compared to the studies on soil animals, relatively limited information is available regarding the effect of MPs on the bioavailability of pesticides to terrestrial plants.

The application of fungicides to control fungal infestations is deemed indispensable to ensure crop production. And fungicide sales have already account for more than 35% of global pesticide sales [19]. However, the behavior and bioavailability of fungicides in the MP-contaminated soil have rarely been explored. The present study therefore aimed to investigate the effect of MPs on the behavior and bioaccumulation of three commonly used fungicides (metalaxyl, azoxystrobin and tebuconazole) in a soil-maize system. PE-MPs, one of the dominant types detected in agricultural soils [20], were selected in this study. The dissipation of the three fungicides in soil as well as their distribution in the tissues of maize (*Zea mays* L.) with and without the amendment of 5% (w/w) PE-MPs were comparatively investigated. Adsorption of the fungicides to soil amended with PE-MPs was also studied to explore its role in pesticide dissipation and bioaccumulation in the soil-maize system. The results will contribute to the risk assessment of co-contamination of microplastics and pesticides in soil.

## Methods and materials

### PE characterization

Low-density PE-MPs were purchased from Yangli Mechanical and Electrical Technology Co., Ltd., Shanghai, China. The morphology and functional groups of the particles were characterized by scanning electron microscopy (SEM, TM-1000, Hitachi Limited, Japan) and Fourier transform infrared spectroscopy (FT-IR, Nicolet is50, Thermo Scientific, USA), respectively.

### Chemicals and soil

Metalaxyl (purity, 99.6%) was obtained from Shenyang Research Institute of Chemical Industry, Shenyang, China. Azoxystrobin (purity, 98.0%) and tebuconazole (purity, 99.3%) were purchased from Shanghai Pesticide Research Institute Co., Shanghai, China. Their physicochemical properties are given in Table S1 in the Supporting Information (SI). HPLC-grade acetonitrile and methanol were supplied by Shanghai Honeywell International, Inc. (Morris Plains, NJ, USA).

The test soil was collected at a depth of 0–20 cm from agricultural land in Lvliang, Shanxi, China. The collected soil sample was air-dried and passed through a 2 mm mesh sieve. The soil physicochemical properties were measured by Zhejiang Jiuan Testing Technology Co., Ltd., and are listed in Table S2. All the pesticides used in this study were not detected in this soil.

### Batch sorption experiments

Batch experiments were performed to acquire the adsorption isotherms of the three fungicides on PE-MPs, soil and their mixture. Each pesticide was dissolved in acetonitrile to form a stock solution, and then diluted with a solution containing 0.01 mol L^−1^ CaCl_2_ and 100 mg L^−1^ NaN_3_ to achieve working solutions at the desired concentrations. The content of acetonitrile in the solution was kept below 0.1% (v/v) to prevent co-solvent effect.

To investigate the adsorption of pesticides by PE-MPs, 0.2 g of PE-MPs were weighed into a centrifuge tube containing 10 mL of working solution at concentrations of 2, 4, 6, 8 and 10 mg L^−1^, respectively, followed by shaking at 150 r/min and 25°C in the dark for 48 h. Our preliminary experiments indicated that equilibrium was reached at 48 h. Subsequently, the subnatant was filtered through a 0.45 µm filter membrane, and the pesticides concentration in the filtrate was detected using high-performance liquid chromatography (HPLC).

Soil adsorption experiment was performed by adding 10 mL of working solutions with different concentrations (1–5 mg L^−1^) into centrifuge tubes with 2 g of soil or soil containing 5% (w/w) PE-MPs. The microplastic concentration used was environmentally relevant based on a previous study, where microplastics at concentrations up to 6.75% (w/w) were detected in soils from an industrial area [21]. After vibration (150 r/min, 25°C) for 48 h, the supernatant was obtained by centrifugation (6000 *g*, 5 min), and subsequently filtered through a 0.45 µm filter membrane for HPLC detection.

### Uptake experiment

After soaking in water for 12 h, maize seeds (*Zea mays L.*) were germinated by wrapping with wet gauze in a seeding tray at 30°C for 2 d. The germinated seeds were then incubated in sterile nutrient solution (pH = 6.5) at 25°C for 7 d in an incubator under irradiation at 250 μmol m^−2^ s^−1^with a photoperiod of 16 h/8 h (light/dark), and a relative humidity of 70%. The nutrient solution was renewed every two days. After 7-d cultivation, seedlings of similar size (plant height of 15 ± 1 cm) were selected and rinsed thoroughly with deionized water.

Each pesticide was mixed thoroughly with soil or soil containing 5% PE-MPs to achieve a final concentration of 5 mg kg^−1^. The selected concentration was similar to the concentration in soil after the application of these pesticides in the field at the recommended dose [22]. Five seedlings were transplanted into each container containing 300 g of the treated soil and cultivated under the abovementioned conditions. Deionized water was supplemented daily to maintain the soil moisture at 60% of the maximum water-holding capacity. The soil was collected at intervals of 0, 7 and 14 d, and the maize plants were harvested at intervals of 7 and 14 d. After thorough rinsing with deionized water and drying with a tissue, the harvested maize plants were sectioned into roots, shoots and leaves. Unplanted soils treated with pesticides and pesticide-free soils with plants were set as controls. The transpiration rate was calculated by subtracting the evaporation of the unplanted controls from the daily weight loss in each treatment. All treatments were performed in triplicate.

### Extraction of pesticides from soil

#### Concentration of pesticides in soil (C_soil_)

Five grams of soil sample (dry weight equivalent) were weighed into a 50 mL centrifuge tube containing 10 mL of acetonitrile and 5 mL of deionized water. After shaking (300 r/min, 10 min) and ultrasonication (20 min), 1 g of NaCl and 4 g of anhydrous MgSO_4_ were added and shaken for 5 min. Afterwards, the tube was centrifuged at 6000 g for 5 min and the supernatant was filtered through a 0.45 µm hydrophilic membrane.

#### Concentration of pesticides in in situ pore water (C_IPW_)

Each soil sample (20 g, wet weight) were weighed into a microsep, which was comprised of a polyethylene centrifuge tube and an inner filter without a filter membrane. After centrifugation (8000 g, 60 min), the in situ pore water was collected from the bottom of the microsep and then filtered through a 0.45 µm hydrophilic membrane for HPLC detection.

#### Concentration of pesticides in ex-situ pore water (C_EPW_)

Soil sample (5 g, dry weight) was mixed with 10 mL of NaN_3_ solution (0.01 mol/L) and shaken at 150 r/min for 48 h. The supernatant was collected by centrifugation (6000 g, 5 min) and filtered through a 0.45 µm hydrophilic membrane prior to HPLC detection.

#### Concentration of pesticides in CaCl_2_ extraction (C_CaCl2_)

Soil sample (2 g, dry weight) was mixed with 10 mL of 0.01 mol/L CaCl_2_ solution containing 100 mg/L NaN_3_. The mixture was shaken in a shaker at 150 r/min for 48 h at 25°CC. After centrifugation at 6000 g for 5 min, the supernatant was collected and filtered through a 0.45 µm hydrophilic membrane for HPLC detection.

### Extraction of pesticides from maize plants

Each tissue sample (2 g) was weighed into a 50 mL centrifuge tube containing 4 mL of deionized water and 8 mL of acetonitrile, and homogenized for 1 min. After shaking for 10 min and ultrasonication for 30 min, 1 g of NaCl and 4 g of MgSO_4_ was added into the tube. The tube was then shaken for 5 min and centrifuged at 6000 g for 5 min. Subsequently, 1.5 mL aliquots of the supernatant were transferred to a 2 mL centrifuge tube containing 10 mg of graphitized carbon black, 50 mg of primary secondary amine and 150 mg of anhydrous MgSO_4_ for purification. Following vortex (30 s) and centrifugation (6000 g, 5 min), the supernatant was passed through a 0.22 µm organic membrane and collected for HPLC analysis.

### HPLC analysis

The analysis were performed on an Agilent 1260 HPLC (Agilent Technologies, USA) equipped with an Agilent Eclipse XDB-C18 column (250 mm × 4.6 mm, 5 μm) and a diode array detector. Detailed detecting conditions are listed in Table S3.

The efficiency of the extraction from soil and maize tissues was verified by recovery experiments with three replicates at three spiked levels. Satisfactory recoveries with relative standard deviations were acquired (Table S4 and S5).

### Data processing and statistical analysis

The Freundlich model (Eq. 1) was adopted to fit the adsorption isotherms:

1 $$C_{\mathrm s}\;=\;K_{\mathrm f}\;\times\;{C_{\mathrm w}}^{1/\mathrm n}$$

where *C*_s_ (mg/kg) is the adsorbed amount; *C*_w_ (mg/L) is the equilibrium concentration; *K*_f_ (mg^1−1/n^ L^1/n^ kg^−1^) and 1/n are the Freundlich adsorption coefficient and the exponential coefficient of the Freundlich model, respectively.

Bioconcentration factors (BCFs), ratios of pesticide concentration (mg kg^−1^) in roots (RCF), stems (SCF) and leaves (LCF) to that in soil (mg kg^−1^), were to evaluate the bioaccumulation of pesticide in maize plants. The translocation ability of fungicides from roots to stems and from stems to leaves was evaluated by translocation factors (TFs). The BCFs and TFs were calculated using the following equations:

2 $$\mathrm{RCF}={\mathrm C}_{\mathrm{root}}/{\mathrm{C}}_\mathrm{soil}$$

3 $$\mathrm{SCF}={\mathrm C}_{\mathrm{stem}}/{\mathrm{C}}_\mathrm{soil}$$

4 $$\mathrm{LCF}={\mathrm C}_{\mathrm{leave}}/{\mathrm{C}}_\mathrm{soil}$$

5 $${\mathrm{TF}}_{\mathrm{stem}/\mathrm{root}}={\mathrm C}_{\mathrm{stem}}/{\mathrm{C}}_\mathrm{root}$$

6 $${\mathrm{TF}}_{\mathrm{leave}/\mathrm{stem}}={\mathrm C}_{\mathrm{leave}}/{\mathrm{C}}_\mathrm{stem}$$

where C_root_, C_stem_ and C_leave_ are the concentrations of three fungicides in the roots, stems, and leaves, respectively.

Each treatment was performed in triplicate. The data are presented as the mean ± standard deviation. Significant differences between samples were analyzed by one-way analysis of variance followed by Duncan post-hoc test (*P* < 0.05) using SPSS 22.0.

## Results and discussion

### Characterization of PE-MPs

Typical SEM images of the PE-MPs are shown in Fig. S1. The PE-MPs had irregular shapes with an average size of 263 μm (*n* = 100). The surfaces of the PE-MPs were relatively smooth, with some protrusions and folds on them. Fig. S2 displays the FT-IR spectra of the PE-MPs. The peaks at 2915, 2848, 1464, and 718 cm^−1^ were attributed to asymmetric C-H stretching, symmetric C-H stretching, -CH_2_ asymmetric bending vibration and -CH_2_ rocking vibration, respectively, which were clearly assigned to the characteristic peaks of PE [23].

### Effect of PE-MPs on the adsorption of the fungicides in soil

**Fig. 1:**
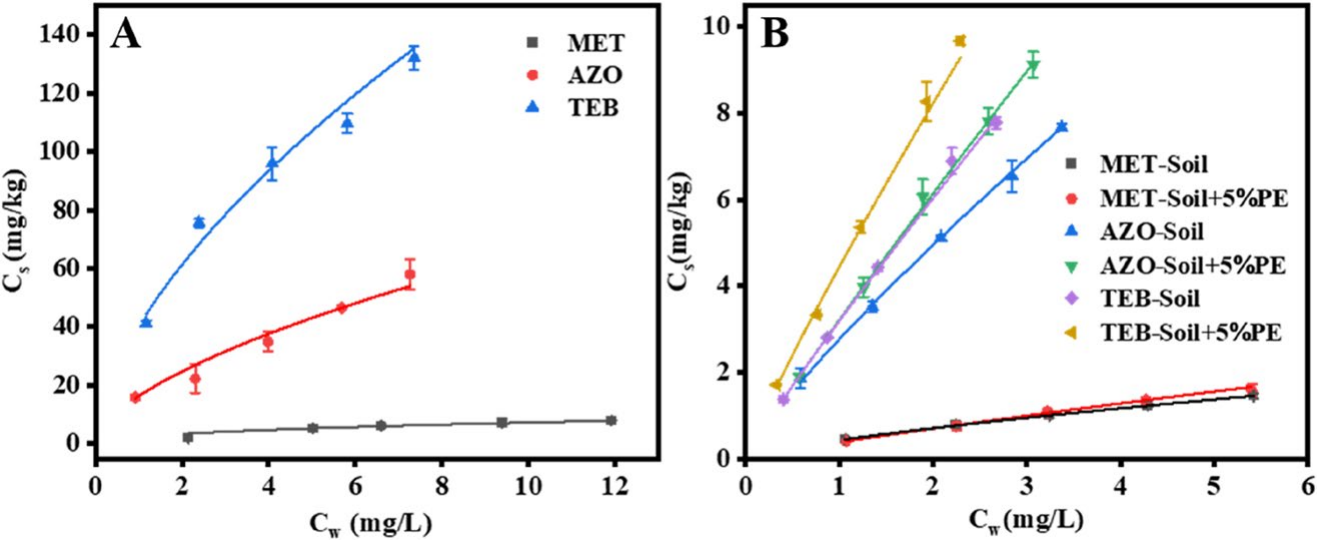
Adsorption isotherms of metalaxyl, azoxystrobin and tebuconazole on PE (**A**) and soils without and with 5% PE-MPs (**B**). MET, metalaxyl; AZO, azoxystrobin; TEB, tebuconazole

Figure 1 illustrates the adsorption isotherms of the three fungicides on PE-MPs, soil and soil-MPs mixture, and the sorption parameters are summarized in Table S6. The correlation coefficients (R^2^) derived from the Freundlich equation were all above 0.94, suggesting that the adsorption isotherms of the pesticides by each adsorbent were well fitted by this model. *K*_f_ values calculated by the Freundlich model reflected the sorption ability of PE-MPs and soil for the pesticides. The adsorption of the fungicides on PE-MPs differed greatly, with *K*_f_ values in the order of tebuconazole > azoxystrobin > metalaxyl (Fig. 1A). Notably, the *K*_f_ values of azoxystrobin and tebuconazole were approximately 6 and 16 times greater than that of metalaxyl, respectively. The adsorption of microplastics for organic chemicals is generally related to the physicochemical properties of the chemical and solution properties. Metalaxyl is a hydrophilic fungicide with high water solubility (8400 mg/L) and a low octanol–water partition coefficient (log*K*_ow_, 1.75); while azoxystrobin and tebuconazole are relatively hydrophobic, with water solubilities of 6.7 and 36 mg/L and log*K*_ow_ of 2.5 and 3.7, respectively. In this study, the log*K*_ow_ of three fungicides was positively related to their *K*_f_ values on PE-MPs (R^2^ = 0.9996), suggesting that pesticides with higher hydrophobicity had a high affinity to PE-MPs. Similar to our results, previous studies reported a positive correlation between the log*K*_ow_ of 5 pesticides (malathion, diflubenzuron, difenoconazole, dipterex and carbendazim) and their *K*_f_ values on PE-MPs derived from agricultural films, and supposed that hydrophobic distribution contributed greatly to the adsorption [24, 25].

The *K*_f_ values for the adsorption of metalaxyl, azoxystrobin and tebuconazole on soil were 0.43 ± 0.01, 2.77 ± 0.03 and 3.19 ± 0.01, respectively, indicating higher adsorption affinity of soil for more hydrophobic pesticides. This was consistent with previous findings that the *K*_f_ values for pesticide adsorption on soil were positively correlated with the log*K*_ow_ of the pesticides [22, 26]. Comparable *K*_f_ value (0.38 ± 0.01) was determined for metalaxyl adsorption on soil-PE mixture; while with the amendment of PE-MPs,* K*_f_ values for the adsorption of azoxystrobin and tebuconazole on soil were increased to 3.22 ± 0.03 and 4.45 ± 0.11, respectively. This indicated that soil with and without PE-MPs showed a comparable and quite low affinity for metalaxyl; whereas the amendment of PE-MPs improved the adsorption ability of soil for azoxystrobin and tebuconazole (Fig. 1B). Although the *K*_f_ value for metalaxyl adsorption on PE-MPs was slightly higher than that on soil, metalaxyl showed a relatively low adsorption affinity to both soil and PE-MPs, and thus the soil adsorption of metalaxyl was barely affected by PE-MPs. Comparatively, PE-MPs had a higher affinity for azoxystrobin and tebuconazole than soil particles as evidenced by much higher *K*_f_ values on PE-MPs, which might be responsible for the enhancement in the adsorption ability of soil by the addition of PE-MPs. At the same pesticides concentration (5 mg L^−1^), the adsorbed amount of azoxystrobin and tebuconazole on soil was increased by 19.0% and 24.4%, respectively, in response to amendment of 5% PE-MPs. Compared to azoxystrobin, PE-MPs had higher adsorption ability for tebuconazole with a higher log*K*_ow_ and were thereby more effective in promoting its adsorption on soil. Similarly, the addition of PE-MPs had a negligible effect on the adsorption of thiacloprid (a hydrophilic insecticide) to soil but facilitated the soil adsorption of phenanthrene, and triclosan (a hydrophobic organic chemicals) have been previously reported [27–29].

### Effect of PE-MPs on the dissipation of the fungicides in unplanted and planted soil

The concentrations of the three fungicides in unplanted and planted soils in the presence and absence of PE-MPs are shown in Fig. S3. Except for metalaxyl in unplanted soils and tebuconazole in MPs amended soils without plantation, the concentrations of the three fungicides generally decreased with time in all the treated soils. The C_soil_ of each fungicide in planted soil was significantly lower than that in unplanted soil, suggesting that the cultivation of maize plants accelerated the dissipation of the three fungicides in soil. This might be ascribed to plant absorption and the biodegradation of pesticides by rhizosphere microorganisms [22]. At 14 d, the residual concentrations of metalaxyl, azoxystrobin and tebuconazole in the planted soil without PE-MPs were 2.86 ± 0.11 mg kg^−1^, 3.44 ± 0.02 mg kg^−1^ and 3.83 ± 0.15 mg kg^−1^, respectively. In the MPs amended soil, the residual concentration of metalaxyl (2.89 ± 0.11 mg kg^−1^) was comparable to that in the MPs-free soil; whereas the residual concentrations of azoxystrobin and tebuconazole decreased to 3.55 ± 0.04 mg kg^−1^ and 4.30 ± 0.07 mg kg^−1^, respectively. The results showed that the dissipation rates varied with pesticides in the order of metalaxyl > azoxystrobin > tebuconazole and that the amendment of 5% PE-MPs prolonged the persistence of azoxystrobin and tebuconazole in soil. Notably, the amendment of PE-MPs led to 3.2% and 12.3% increases in C_soil_ of azoxystrobin and tebuconazole at 14 d, respectively, indicating that PE-MPs were more effective in decelerating the dissipation of tebuconazole. Similar results have been reported in previous studies, where polystyrene (PS) and polyvinyl chloride (PVC) microplastics exhibited negligible influence on the dissipation of thiacloprid [27], and PE-MPs inhibited the removal of the hydrophobic compound phenanthrene from soil [30].

The water-soluble portions (C_CaCl2_, C_EPW_ and C_IPW_) of pesticides are generally considered as bioavailable fractions in soil [31]. Therefore, the C_CaCl2_, C_EPW_ and C_IPW_ of the three fungicides in the planted soil with and without PE-MPs were measured. As evident in Table 1, no significant difference was observed in the C_CaCl2_, C_EPW_ and C_IPW_ of metalaxyl between the treatments with and without PE-MPs. In contrast to those of C_soil_, the C_CaCl2_, C_EPW_ and C_IPW_ of azoxystrobin and tebuconazole were much lower than those of metalaxyl. The amendment of PE-MPs decreased the C_CaCl2_, C_EPW_ and C_IPW_ of azoxystrobin and tebuconazole at 7 and 14 d, with the reduction in C_IPW_ being the most remarkable. Specifically, the C_IPW_ of azoxystrobin and tebuconazole was respectively reduced by 23.9% and 45.5%, respectively, at 7 d and by 16.7% and 59.0%, respectively, at 14 d, showing that the amendment of PE-MPs caused greater reductions in the bioavailability of tebuconazole.

**Table 1:** Pesticide concentrations in planted soil extracted by different methods

Pesticides	Exposure time (d)	Treatments	C_soil_ (mg kg^−1^)	C_CaCl2_ (mg L^−1^)	C_IPW_ (mg L^−1^)	C_EPW_ (mg L^−1^)
**Metalaxyl**	7	Soil	3.69 ± 0.15^a^	0.78 ± 0.03^a^	0.95 ± 0.03^a^	1.82 ± 0.02^a^
		Soil + 5% PE	3.69 ± 0.05^a^	0.79 ± 0.02^a^	0.96 ± 0.05^a^	1.81 ± 0.03^a^
	14	Soil	2.86 ± 0.11^a^	0.71 ± 0.04^a^	0.93 ± 0.01^a^	1.56 ± 0.03^a^
		Soil + 5% PE	2.89 ± 0.11^a^	0.70 ± 0.04^a^	0.93 ± 0.01^a^	1.59 ± 0.10^a^
**Azoxystrobin**	7	Soil	3.64 ± 0.05^a^	0.44 ± 0.04^a^	0.71 ± 0.04^b^	0.70 ± 0.02^a^
		Soil + 5% PE	3.72 ± 0.11^a^	0.36 ± 0.01^a^	0.54 ± 0.05^a^	0.57 ± 0.02^b^
	14	Soil	3.44 ± 0.02^b^	0.24 ± 0.02^a^	0.60 ± 0.07^a^	0.35 ± 0.07^a^
		Soil + 5% PE	3.55 ± 0.02^a^	0.19 ± 0.02^a^	0.50 ± 0.02^a^	0.28 ± 0.01^a^
**Tebuconazole**	7	Soil	4.29 ± 0.05^b^	0.28 ± 0.04^a^	0.88 ± 0.17^b^	0.55 ± 0.04^a^
		Soil + 5% PE	4.53 ± 0.03^a^	0.21 ± 0.01^b^	0.48 ± 0.08^a^	0.44 ± 0.03^b^
	14	Soil	3.83 ± 0.15^b^	0.28 ± 0.03^a^	0.78 ± 0.13^b^	0.40 ± 0.03^a^
		Soil + 5% PE	4.30 ± 0.07^a^	0.21 ± 0.02^b^	0.32 ± 0.04^a^	0.36 ± 0.01^a^

Different letters indicate significant difference between treatments for the same pesticide at the same time (*P* < 0.05)

Pesticides in soil could be degraded through abiotic and biological processes (e.g., hydrolysis, photolysis, oxidation, biodegradation, etc.) [32]. It is well documented that the water-soluble portion of organic chemicals was more susceptible to biodegradation since it was more bioavailable to environmental microorganisms than the portion that was tightly adsorbed on soil constituents [33]. The enhancement of soil adsorption for azoxystrobin and tebuconazole, facilitated by PE-MPs, led to a decline in their water-soluble portion in soil. This reduction consequently lessened the biodegradation, which may partially account for the inhibitory effect of PE-MPs on their dissipation. Compared to azoxystrobin, PE-MPs were more effective in enhancing the soil adsorption for tebuconazole, resulting in a more significant decrease in its bioavailable fraction, and subsequently inhibiting its dissipation more effectively. However, PE-MPs had a negligible effect on the bioavailability of metalaxyl due to the comparable adsorption ability of soil with and without the amendment of PE-MPs, and as a result, its dissipation remained unaffected.

### Effect of PE-MPs on the accumulation of the fungicides in maize plants

There was no significant difference in the plant heights, fresh weights and transpiration rates of the maize seedlings between the treatments and controls (Fig. S4), indicating that both the three fungicides and PE-MPs at the fortified concentrations had no obvious phytotoxicity.

**Fig. 2:**
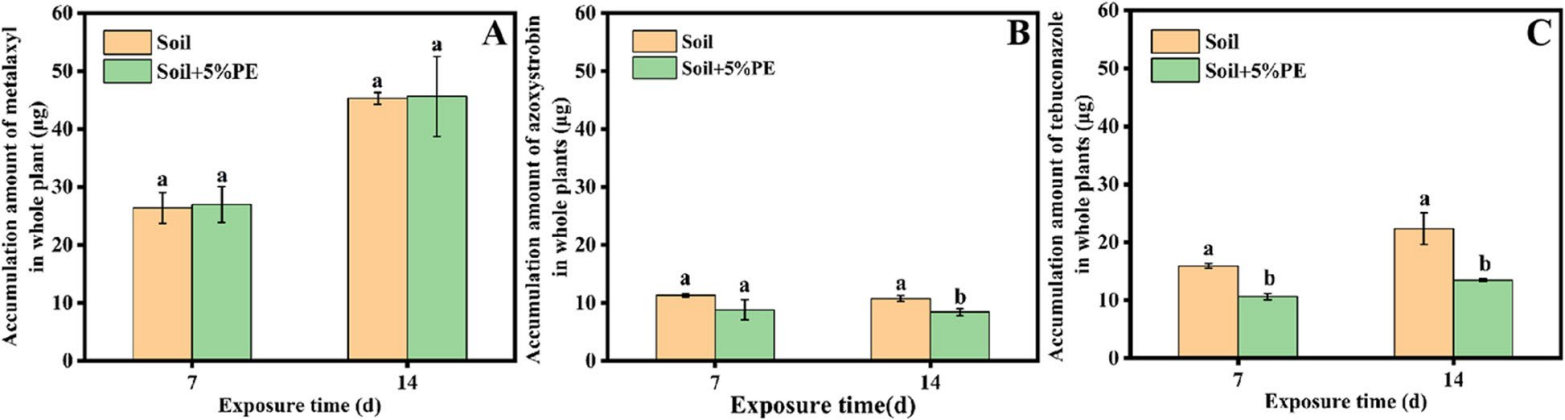
Accumulation amount of metalaxyl (**A**), azoxystrobin (**B**) and tebuconazole (**C**) in whole plant. Error bars represent standard deviations (*n* = 3). Different letters indicate significant differences between different treatments at the same time (*P* < 0.05)

The amount of the fungicides in whole plant of maize grown in soils in the presence and absence of PE-MPs is illustrated in Fig. 2. The accumulation amount of metalaxyl in the whole plant of maize grown in soil with and without PE-MPs were 26.96 ± 3.07 and 26.40 ± 2.68 μg, respectively, at 7 d and sharply increased to 45.65 ± 6.9 and 45.31 ± 1.02 μg, respectively, at 14 d (Fig. 2A). Therefore, amendment of PE-MPs had little effect on the accumulation of metalaxyl in maize. In comparison, substantially less azoxystrobin and tebuconazole accumulated in maize plants at the same fortified concentration (5 mg kg^−1^) (Fig. 2B and C). The accumulation amount of azoxystrobin in the maize planted in MPs free soil was 11.31 ± 0.29 μg at 7 d and maintained almost constant at 14 d, which were reduced by approximately 22% in the soil amended with PE-MPs at both 7 and 14 d. The amount of tebuconazole in the maize planted in the soil without PE-MPs was 15.92 ± 0.42 μg at 7 d and increased to 22.34 ± 2.7 μg at 14 d, while the amendment of PE-MPs caused 33.2% and 39.7% reductions in tebuconazole accumulation at 7 and 14 d, respectively. Residual pesticides may enter the plant through root uptake of the bioavailable fraction in soil, which strongly depends on soil adsorption. And C_IPW_ was considered an optimum candidate for estimating bioavailability as compared to C_CaCl2_ and C_EPW_ [22, 26]. Accordingly, the decreased accumulation of azoxystrobin and tebuconazole in maize plants with the amendment of PE-MPs might be attributable to the reduced bioavailability resulted from their enhanced adsorption on soil. Moreover, PE-MPs induced a more significant decrease in the bioavailability of tebuconazole and thus inhibited its accumulation more effectively. Similarly, polystyrene microplastics were previously found to decrease the uptake of phenanthrene in soybean [18]. Wang et al. [34] reported that PE-MPs at amendment rates of 1–10% increased the soil sorption for phenanthrene, fluoranthene and pyrene, resulting in a significant reduction in the *C*_free_ in soil pore water and thus reducing their accumulation by earthworms.

### Effect of PE-MPs on the uptake and translocation of the fungicides in maize plants

**Fig. 3:**
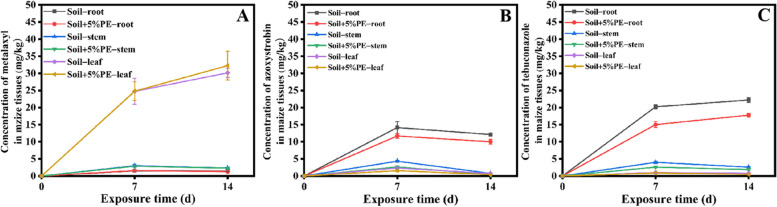
Concentrations of metalaxyl (**A**), azoxystrobin (**B**) and tebuconazole (**C**) in maize tissues. Error bars represent standard deviations (*n* = 3)

Figure 3 shows the concentration of each fungicide in different tissues of maize. In the MPs-free treatment, metalaxyl concentration in roots and stems were 1.52 ± 0.24 and 3.00 ± 0.40 mg kg^−1^ at 7 d, respectively, and then slightly decreased to 1.46 ± 0.10 and 2.32 ± 0.08 mg kg^−1^ at 14 d, respectively. And metalaxyl concentration in maize leaves increased from 24.74 ± 3.77 mg kg^−1^ at 7 d to 30.14 ± 1.33 mg kg^−1^ at 14 d, which were much higher than those in roots and stems. This indicated that matalaxyl was easily upward translocated and primarily accumulated in maize leaves. No significant differences were observed in metalaxyl concentration in each tissue between the treatments with and without PE-MPs at the same time. In soil without PE-MPs, azoxystrobin concentrations in roots, shoots and leaves rapidly increased to 14.16 ± 1.75, 4.32 ± 0.24 and 2.33 ± 0.25 mg kg^−1^ at 7 d, respectively, and then declined to 11.71 ± 0.72, 2.65 ± 0.26 and 1.56 ± 0.29 mg kg^−1^ at 14 d, respectively, suggesting that azoxystrobin was more prone to accumulation in maize root. As compared to the soil-only group, azoxystrobin concentration were significantly lower (*P* < 0.05, except for the root concentration at 7 d) in the tissues of maize plants grown in soil amended with PE-MPs. Tebuconazole concentration in roots in treatments without and with PE-MPs dramatically increased to 20.23 ± 0.67 and 15.03 ± 0.87 mg kg^−1^ within 7 d, and then gradually increased to 22.21 ± 0.70 and 17.79 ± 0.55 mg kg^−1^ at 14 d, respectively. Similar to the case of azoxystrobin, the concentrations of tebuconazole in stems and leaves were much lower than those in roots in MPs-free treatment with the values of 4.00 ± 0.37 and 1.00 ± 0.24 mg kg^−1^ at 7 d and 2.59 ± 0.20 and 0.78 ± 0.17 mg kg^−1^ at 14 d, respectively. The amendment of PE-MPs slightly reduced the tebuconazole concentration in maize stems but hardly affect its accumulation in leaves. The results clearly showed that the absorbed metalaxyl was dominantly located in leaves while azoxystrobin and tebuconazole were mainly distributed in roots of maize. Previous studies have proposed that pesticides with lower log*K*_ow_ and higher *S*_w_ are more readily translocated upward via transpiration and thus accumulated in leaves; while hydrophobic pesticides have a higher affinity for the lipophilic constituent of root solids and are thus retained in roots [22]. This may explain the contrasting distribution patterns for metalaxyl, azoxystrobin and tebuconazole in maize plants. Similarly, Kubicki et al. [35] found that nearly 70% of metalaxyl was located in the leaves of tomato plants. And significantly higher concentrations of azoxystrobin and tebuconazole in roots than in shoots were also previously observed in maize plants [22]. Since azoxystrobin and tebuconazole were mainly accumulated in maize roots, the PE-MPs induced the greatest changes in their concentrations in roots, where concentrations of azoxystrobin and tebuconazole were reduced by 17.3–17.5% and 19.9–25.7%, respectively.

**Fig. 4:**
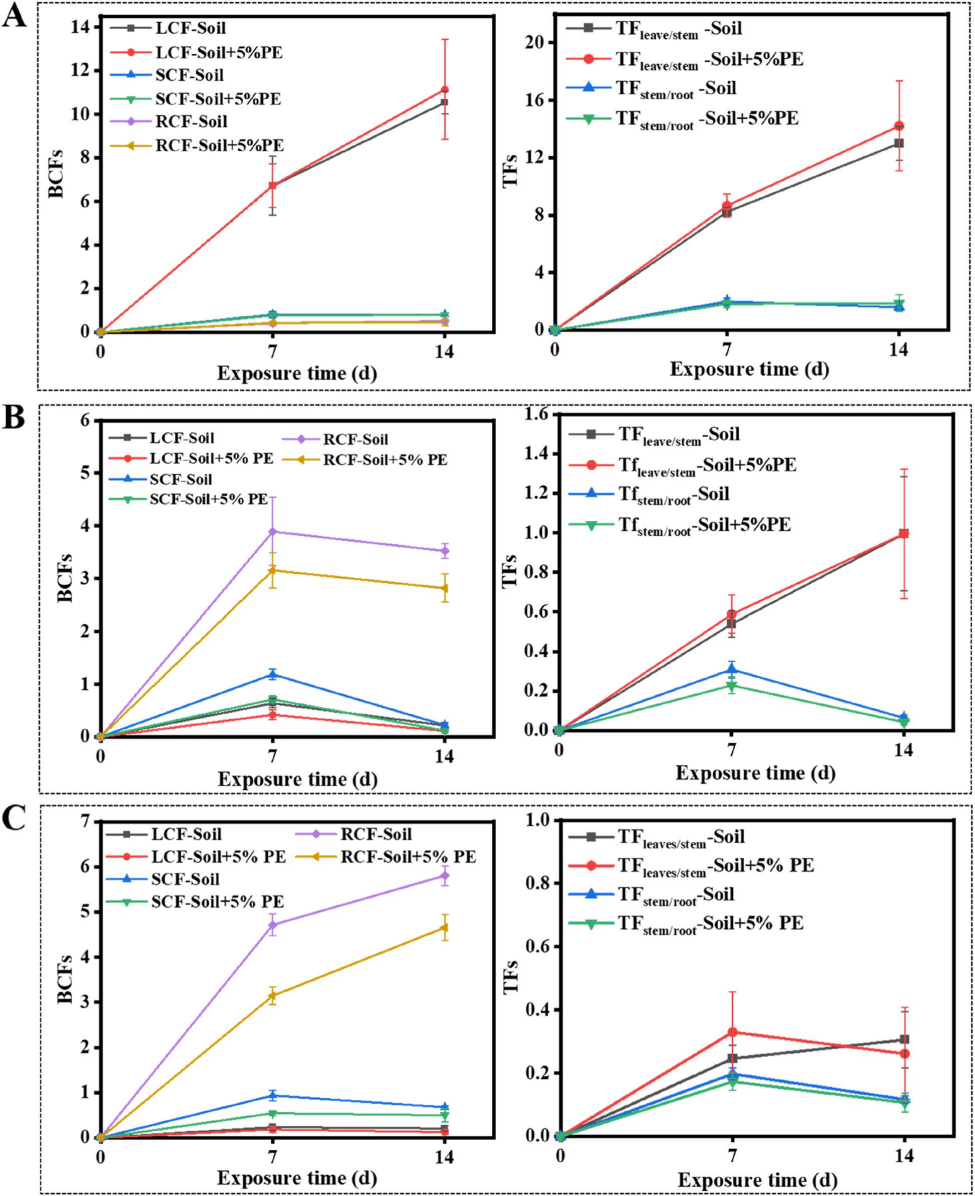
BCFs and TFs of metalaxyl (**A**), azoxystrobin (**B**) and tebuconazole (**C**) in maize. Error bars represent standard deviations (*n* = 3)

BCFs and TFs were determined for estimating the effect of PE-MPs on the capability of maize to absorb and translocate the pesticides. As shown in Fig. 4A, the LCF of metalaxyl increased with time, reaching the levels of 10.5 ± 0.5 and 11.3 ± 2.3 in treatments without and with PE-MPs, respectively, at 14 d. The LCF values of metalaxyl were much greater than SCF and RCF regardless of PE-MP amendment. And the BCFs of metalaxyl in maize grown in soil amended with PE-MPs were comparable to those in unamended soil. The TF values for metalaxyl from root to stem and stem to leaf were both greater than 1, indicating a high capability of maize to translocate metalaxyl from roots to leaves. Conversely, the RCF values of azoxystrobin and tebuconazole were significantly greater than SCF and LCF (Fig. 4B and C). With the amendment of PE-MPs, the RCF of azoxystrobin at 14 d decreased from 3.5 ± 0.1 to 2.8 ± 0.3, and that of tebuconazole decreased from 5.8 ± 0.2 to 4.6 ± 0.3. The TF_stem/root_ and TF_leave/stem_ for both fungicides were all less than 1, suggesting limited translocation of them from roots to the leaves (Fig. 4D and F). Despite the variations in the BCFs of the three fungicides with the amendment of PE-MPs, the TFs for each fungicide were almost unaffected by PE-MPs. Previous studies have reported that the high affinity of microplastics for organic pollutants, especially those with high hydrophobicity enabled them to act as a “Trojan horse” and thus promoted the bioaccumulation of the pollutants [36]. An earlier study found that polystyrene and polymethylmethacrylate particles (0.2 and 2 μm) were absorbed by crop plants via a crack-entry mode and transported from roots to shoots [37]. However, the PE-MPs used in the present study could hardly penetrate the roots of the maize plant due to their large size, and therefore had negligible effect on the translocation of the three fungicides in maize plants.

## Conclusion

In this study, the influence of PE-MPs on the accumulation of three fungicides (metalaxyl, azoxystrobin and tebuconazole) in maize was explored. The results from the adsorption experiments showed that hydrophobic distribution played a crucial role in the adsorption behaviors of the fungicides on PE-MPs. PE-MPs exhibited relatively weak adsorption ability for the hydrophilic fungicide metalaxyl, and thus had little impact on its adsorption to soil; whereas PE-MPs showed much greater adsorption affinity for the hydrophobic pesticides (azoxystrobin and tebuconazole) than soil, and thus promoted their adsorption to soil. The enhancement in soil adsorption of azoxystrobin and tebuconazole with the addition of 5% PE-MPs reduced their bioavailable fractions in soil, especially C_IPW_, thereby slowing down their dissipation in soil and reducing their accumulation in maize plants. More importantly, PE-MPs had a greater impact on the adsorption of tebuconazole to soil compared to azoxystrobin, resulting in higher degree of decrease in tebuconazole dissipation and bioaccumulation. Nevertheless, PE-MPs had negligible effect on the accumulation of metalaxyl in maize since metalaxyl adsorption to soil was unaffected. Moreover, the translocation of each fungicide from roots to leaves were hardly affected with the amendment of PE-MPs, excluding the possibility of PE-MPs as vectors to deliver the fungicides into plant tissues. Despite the reduced pesticide bioaccumulation, the prolonged environmental persistence of pesticide when MPs co-exist should be great concern.

## Supplementary Information

Additional file 1: Table S1. Physicochemical properties of the tested fungicides in this study. Table S2. Physicochemical properties of the tested soil. Table S3. HPLC analysis conditions of the tested fungicides in this study. Table S4. Recoveries and RSD values for the tested fungicides in soil. Table S5. Recoveries and RSD values for the tested fungicides in maize tissues. Table S6. Adsorption parameters of the tested fungicides by different sorbents. Fig. S1. Typical SEM images of PE-MPs. Fig. S2. FT-IR spectrum of PE-MPs. Fig. S3. Concentrations of metalaxyl (A), azoxystrobin (B) and tebuconazole (C) in planted and unplanted soil. Error bars represent standard deviations (*n* = 3). Fig. S4. The plant height, weight and transpiration rate of maize planted in soil treated with metalaxyl (A), azoxystrobin (B) and tebuconazole (C). Images in each panel from left to right show plant height, plant weight and transpiration rate of maize, respectively. Error bars represent standard deviations (*n* = 3). Values with the same letter (a) at the same time indicate no significant difference among different treatments.

## Data Availability

The data will be made available on reasonable request.
