# Supplementary material for: Different effects of polyethylene microplastics on bioaccumulation of three fungicides in maize (Zea mays L.)
Source: Crop Health. 2024 May 21;2(1):7. doi: 10.1007/s44297-024-00028-x (PMC12825916; doi:10.1007/s44297-024-00028-x)
Supplement: Supplementary file 1 — Additional file 1: Table S1. Physicochemical properties of the tested fungicides in this study. Table S2. Physicochemical properties of the tested soil. Table S3. HPLC analysis conditions of the tested fungicides in this study. Table S4. Recoveries and RSD values for the tested fungicides in soil. Table S5. Recoveries and RSD values for the tested fungicides in maize tissues. Table S6. Adsorption parameters of the tested fungicides by different sorbents. Fig. S1. Typical SEM images of PE-MPs. Fig. S2. FT-IR spectrum of PE-MPs. Fig. S3. Concentrations of metalaxyl (A), azoxystrobin (B) and tebuconazole (C) in planted and unplanted soil. Error bars represent standard deviations (n = 3). Fig. S4. The plant height, weight and transpiration rate of maize planted in soil treated with metalaxyl (A), azoxystrobin (B) and tebuconazole (C). Images in each panel from left to right show plant height, plant weight and transpiration rate of maize, respectively. Error bars represent standard deviations (n = 3). Values with the same letter (a) at the same time indicate no significant difference among different treatments. [file 44297_2024_28_MOESM1_ESM.docx]

**S****upporting Information (SI)**

**Different effects of polyethylene microplastics on bioaccumulation of three fungicides in maize (*Zea mays* L.)**

Shuimin Qiu, Hongjian Shen, Jialu Song, Hua Fang, Yunlong Yu, Luqing Zhang^*^

Institute of Pesticide and Environmental Toxicology, the Key Laboratory of Molecular Biology of Crop Pathogens and Insects, the Key Laboratory of Biology of Crop Pathogens and Insects of Zhejiang Province, College of Agricultural and Biotechnology, Zhejiang University, Hangzhou 310058, China.

*Corresponding author: zhangluqing@zju.edu.cn

**Table S1. Physicochemical properties of the tested fungicides in this study**

| **Pesticides** | **log *K*_ow_** | **Water solubility (mg L^-1^)** | **p*K*_a_** | **Molecular weight** |
| --- | --- | --- | --- | --- |
| **Metalaxyl** | 1.75 | 8400 | 0 | 279.3 |
| **Azoxystrobin** | 2.5 | 6.7 | -0.93 | 403.4 |
| **Tebuconazole** | 3.7 | 36 | 5 | 307.82 |

**Table S2. Physicochemical properties of the tested soil**

| **OMC (%)** | **pH** | **CEC (cmol kg^-1^)** | **Total N (%)** | **Total P (%)** | **Silt (%)** | **Clay (%)** | **Sand (%)** | **Texture** |
| --- | --- | --- | --- | --- | --- | --- | --- | --- |
| 0.4 | 8.72 | 8.6 | 0.02 | 0.05 | 47.3 | 20.3 | 32.5 | loam |

OMC, organic matter content; CEC, cation exchange capacity.

**Table S3. HPLC analysis conditions of the tested fungicides in this study**

| **Pesticides** | **Flow rate**  **(mL min^-1^)** | **Injection volume (μL)** | **Liquid phase**  **(v:v)** | **Column temperature**  **(°C)** | **Detection wavelength**  **(nm)** |
| --- | --- | --- | --- | --- | --- |
| **Metalaxyl** | 1.0 | 10 | Acetonitrile: methanol: water  30:30:40 | 25 | 215 |
| **Azoxystrobin** | 1.0 | 10 | Acetonitrile: water  60:40 | 30 | 254 |
| **Tebuconazole** | 1.0 | 10 | Acetonitrile: water  75:25 | 30 | 220 |

**Table S4. Recoveries and RSD values for the tested fungicides in soil**

| **Pesticides** | **Matrices** | **Spiked level**  **(mg kg^-1^)** | **Recovery**  **(%)** | **RSD**  **(%)** |
| --- | --- | --- | --- | --- |
| **Metalaxyl** | Soil | 0.1 | 91.4 | 0.7 |
|  |  | 1 | 88.5 | 8.5 |
|  |  | 5 | 100.9 | 4.8 |
|  | Soil+5% PE-MPs | 0.1 | 97.7 | 0.5 |
|  |  | 1 | 90.5 | 6.5 |
|  |  | 5 | 98.2 | 3.7 |
| **Azoxystrobin** | Soil | 0.1 | 92.5 | 1.0 |
|  |  | 1 | 96.3 | 1.9 |
|  |  | 5 | 93.4 | 1.8 |
|  | Soil+5% PE-MPs | 0.1 | 93.8 | 1.1 |
|  |  | 1 | 96.8 | 1.9 |
|  |  | 5 | 96.8 | 2.4 |
| **Tebuconazole** | Soil | 0.1 | 113.8 | 1.4 |
|  |  | 1 | 87 | 2.6 |
|  |  | 5 | 97.8 | 0.6 |
|  | Soil+5% PE-MPs | 0.1 | 114.5 | 1.5 |
|  |  | 1 | 85.5 | 1.5 |
|  |  | 5 | 96.5 | 0.8 |

**Table S5. Recoveries and RSD values for the tested fungicides in maize tissues**

| **Pesticides** | **Tissues** | **Spiked level (mg/kg)** | **Recovery (%)** | **RSD (%)** |
| --- | --- | --- | --- | --- |
| **Metalaxyl** | roots | 1 | 99.4 | 10.4 |
|  |  | 5 | 111.6 | 2.0 |
|  |  | 10 | 107.4 | 5.3 |
|  | stems | 1 | 99.4 | 2.0 |
|  |  | 5 | 102.7 | 0.9 |
|  |  | 10 | 109.1 | 4.8 |
|  | leaves | 1 | 101.1 | 10.3 |
|  |  | 5 | 105.2 | 2.1 |
|  |  | 10 | 107.6 | 0.3 |
| **Azoxystrobin** | roots | 0.1 | 90.3 | 0.8 |
|  |  | 1 | 80.4 | 1.0 |
|  |  | 5 | 99.8 | 2.1 |
|  | stems | 0.1 | 93.0 | 1.0 |
|  |  | 1 | 95.2 | 3.8 |
|  |  | 5 | 103.7 | 9.9 |
|  | leaves | 0.1 | 93.8 | 0.4 |
|  |  | 1 | 94.7 | 2.9 |
|  |  | 5 | 96.3 | 3.1 |
| **Tebuconazole** | roots | 0.1 | 100.3 | 0.8 |
|  |  | 1 | 104.4 | 5.8 |
|  |  | 5 | 95.2 | 5.8 |
|  | shoots | 0.1 | 113.6 | 1.6 |
|  |  | 1 | 100.2 | 5.0 |
|  |  | 5 | 104.3 | 4.5 |
|  | leaves | 0.1 | 106.9 | 1.1 |
|  |  | 1 | 98.6 | 2.9 |
|  |  | 5 | 111.3 | 3.9 |

**Table S6. Adsorption parameters of the tested fungicides by different sorbents**

| **Pesticides** | **Sorbents** | **Adsorption parameters** | | |
| --- | --- | --- | --- | --- |
|  |  | ***K*_f_** | **1/n** | **R^2^** |
| **Metalaxyl** | PE-MPs | 2.51 ± 0.31 | 0.46 ± 0.05 | 0.962 |
|  | Soil | 0.43 ± 0.01 | 0.73 ± 0.01 | 0.999 |
|  | Soil+5% PE-MPs | 0.38 ± 0.01 | 0.88 ± 0.02 | 0.997 |
| **Azoxystrobin** | PE-MPs | 16.36 ± 0.89 | 0.60 ± 0.03 | 0.996 |
|  | Soil | 2.77 ± 0.03 | 0.84 ± 0.01 | 1.000 |
|  | Soil+5% PE-MPs | 3.22 ± 0.03 | 0.93 ± 0.01 | 1.000 |
| **Tebuconazole** | PE-MPs | 40.30 ± 3.19 | 0.61 ± 0.06 | 0.948 |
|  | Soil | 3.19 ± 0.01 | 0.92 ± 0.01 | 0.999 |
|  | Soil+5% PE-MPs | 4.45 ± 0.11 | 0.89 ± 0.03 | 0.991 |


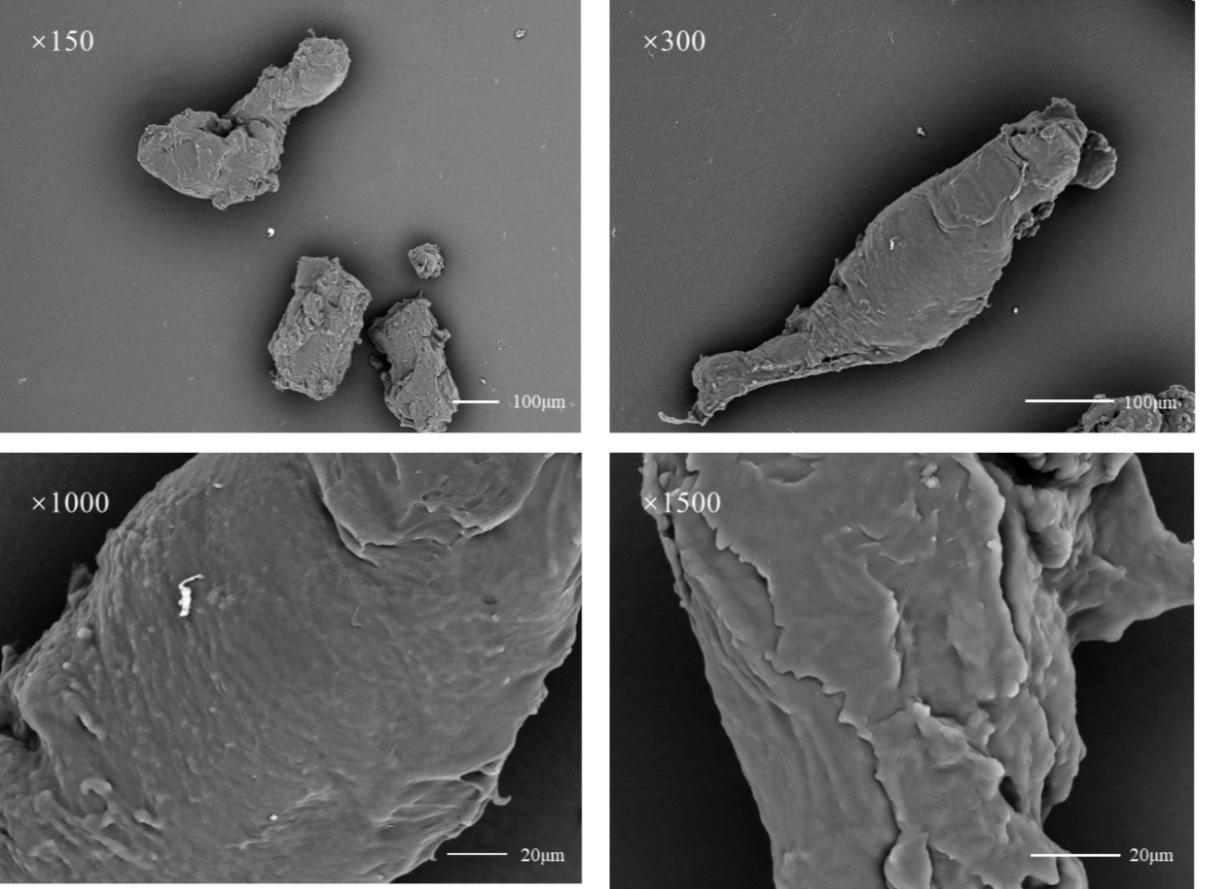


**Fig. S1** Typical SEM images of PE-MPs


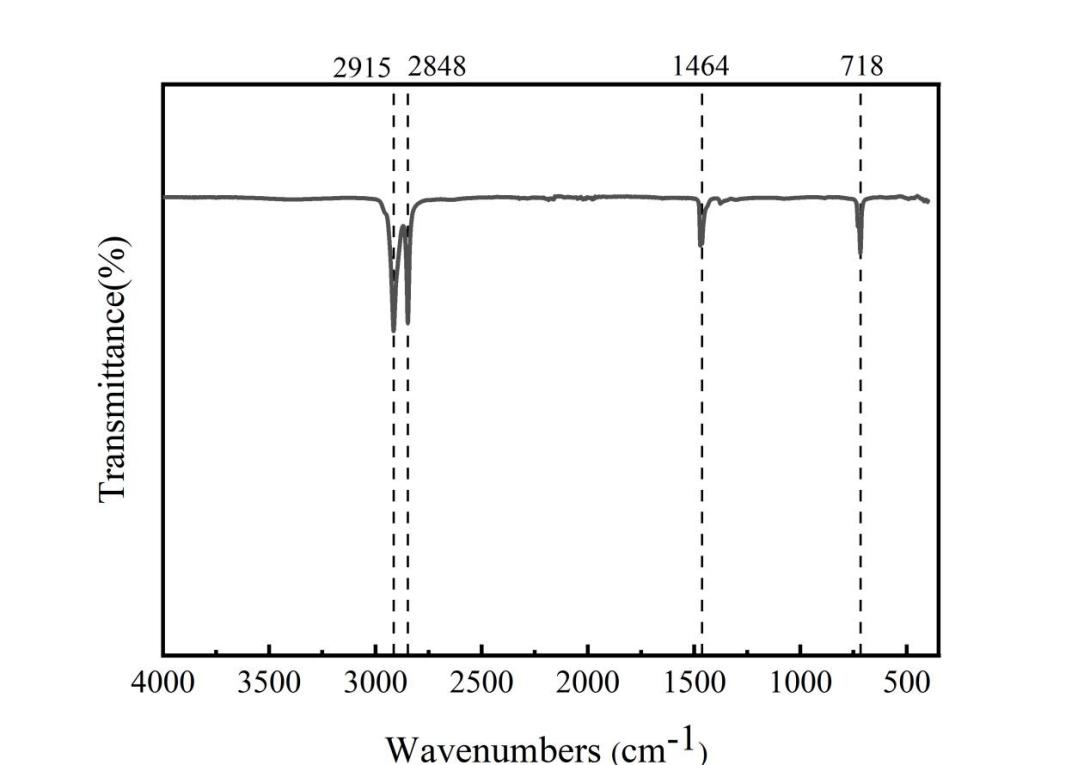


**Fig. S2** FT-IR spectrum of PE-MPs

**
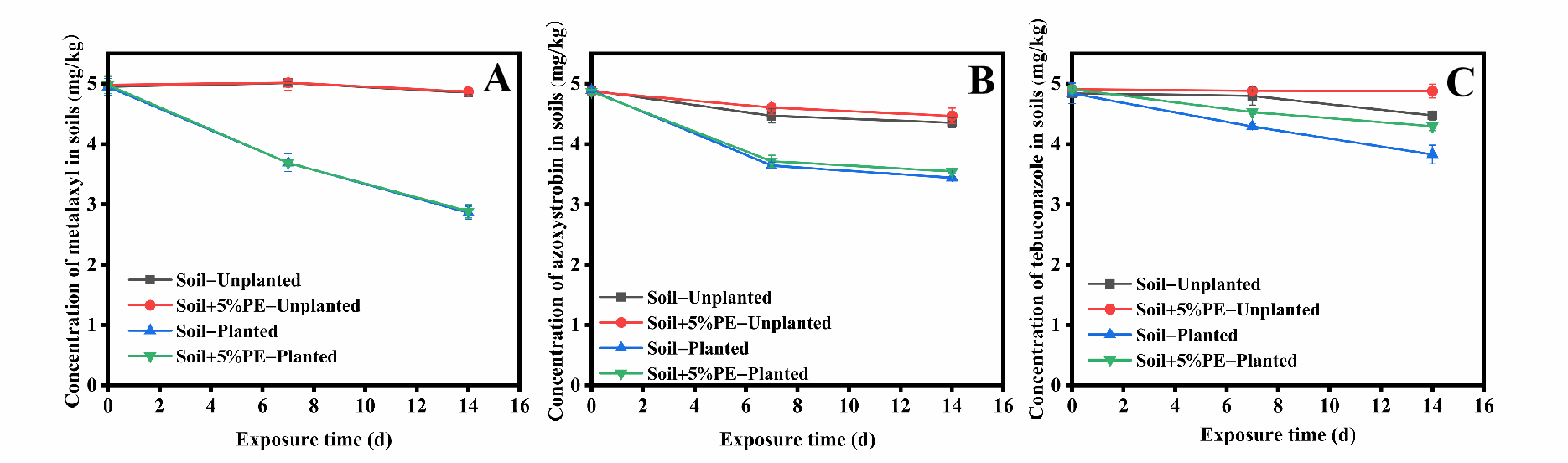
**

**Fig. S3** Concentrations of metalaxyl (**A**), azoxystrobin (**B**) and tebuconazole (**C**) in planted and unplanted soil. Error bars represent standard deviations (n = 3)

**
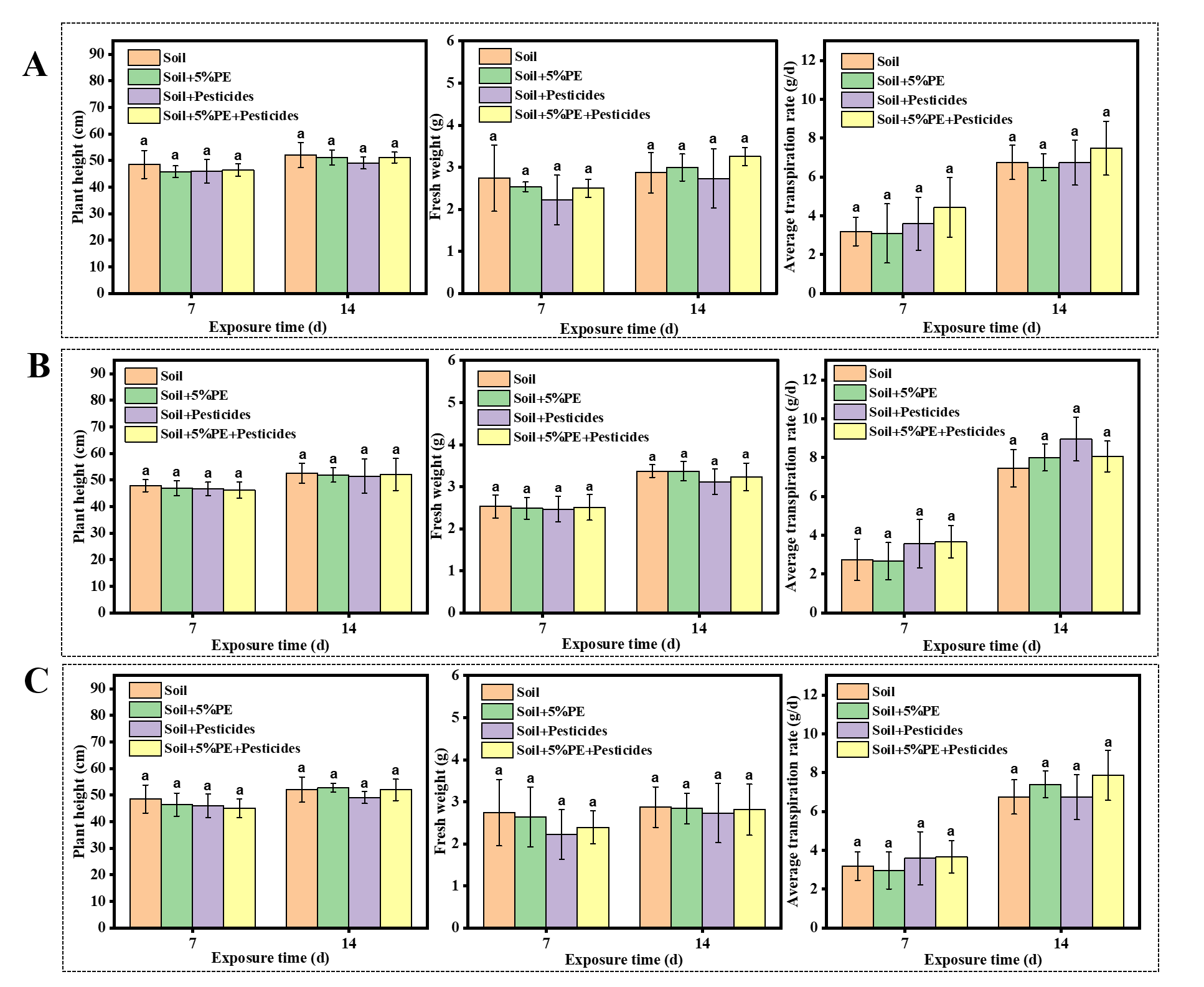
Fig. S4** The plant height, weight and transpiration rate of maize planted in soil treated with metalaxyl (**A**), azoxystrobin (**B**) and tebuconazole (**C**). Images in each panel from left to right show plant height, plant weight and transpiration rate of maize, respectively. Error bars represent standard deviations (n = 3). Values with the same letter (a) at the same time indicate no significant difference among different treatments
